# The epidemiology of AIDS-associated non-Hodgkin's lymphoma in the World Health Organization European Region.

**DOI:** 10.1038/bjc.1992.384

**Published:** 1992-11

**Authors:** D. Serraino, G. Salamina, S. Franceschi, D. Dubois, C. La Vecchia, J. B. Brunet, R. A. Ancelle-Park

**Affiliations:** Epidemiology Unit, Aviano Cancer Centre, Italy.

## Abstract

This paper describes the epidemiology of AIDS-associated non-Hodgkin's lymphoma (NHL) in the World Health Organization (WHO) European Region. Data, collected by the WHO Collaborating Centre on AIDS in Paris, France, were derived from the national AIDS surveillance systems of 21 countries. Among 53,042 cases reported as of the end of June 1991, 1,617 (3.0%) had NHL as the presenting clinical manifestation of AIDS. The proportion of cases presenting with NHL ranged from 1.1% in children infected perinatally to 3.9% among haemophiliacs. In comparison with intravenous drug users (IVDUs) (2.6% of whom had NHL), a moderate excess was found among homosexual or bisexual men (odds ratio - OR -:1.2, 95% confidence interval - CI -:1.0-1.3). Over time, the proportion of NHL was constant, but whereas among homosexual or bisexual men the frequency of NHL as AIDS-indicator disease significantly increased (9.7% per year), among IVDUs a significant downward trend emerged (17.1% per year). In respect to age, two peaks of NHL were seen at the age groups 10-19 (3.8%) and 50-59 (4.3%). The proportion of AIDS-associated NHL significantly increased with increasing age among homosexual and bisexual men and heterosexuals whereas it decreased among IVDUs. All these differences, however, have to be interpreted cautiously on account of the limitations of the reporting systems.


Br. J. Cancer (1992), 66, 912-916 © Macmillan Press Ltd., 1992

### The epidemiology of AIDS-associated non-Hodgkin's lymphoma in the World Health Organization European Region

D. Serraino[1], G. Salamina[2], S. Franceschi[1], D. Dubois[2], C. La Vecchia[3,4], J.B. Brunet[2] & R.A. Ancelle-Park[2]

[1]*Epidemiology Unit, Aviano Cancer Centre, 33081 Aviano (PN), Italy;* [2]*World Health Organization - European Community Collaborating Centre on AIDS, Hopital National de Saint-Maurice, 94410 Paris, France;* [3]*'Mario Negri' Institute for Pharmacological Research, 20157 Milan, Italy;* [4]*Institute of Social and Preventive Medicine, University of Lausanne, 1500 Lausanne, Switzerland.*

**Summary** This paper describes the epidemiology of AIDS-associated non-Hodgkin's lymphoma (NHL) in the World Health Organization (WHO) European Region. Data, collected by the WHO Collaborating Centre on AIDS in Paris, France, were derived from the national AIDS surveillance systems of 21 countries. Among 53,042 cases reported as of the end of June 1991, 1,617 (3.0%) had NHL as the presenting clinical manifestation of AIDS. The proportion of cases presenting with NHL ranged from 1.1% in children infected perinatally to 3.9% among haemophiliacs. In comparison with intravenous drug users (IVDUs) (2.6% of whom had NHL), a moderate excess was found among homosexual or bisexual men (odds ratio - OR -:1.2, 95% confidence interval - CI -:1.0-1.3). Over time, the proportion of NHL was constant, but whereas among homosexual or bisexual men the frequency of NHL as AIDS-indicator disease significantly increased (9.7% per year), among IVDUs a significant downward trend emerged (17.1% per year). In respect to age, two peaks of NHL were seen at the age groups 10-19 (3.8%) and 50-59 (4.3%). The proportion of AIDS-associated NHL significantly increased with increasing age among homosexual and bisexual men and heterosexuals whereas it decreased among IVDUs. All these differences, however, have to be interpreted cautiously on account of the limitations of the reporting systems.

Non-Hodgkin's lymphoma (NHL) is a group of heterogeneous malignancies representing, in Western countries, less than 2% of all neoplasms in the general population (Levi *et al.*, 1989; Jensen *et al.*, 1990). In Europe, the highest incidence rates for NHL were registered in Switzerland and Northern Italy among males, and in Scotland and Switzerland among females. Eastern European countries showed lower rates than Western countries (Levi *et al.*, 1989).

It is known that the risk of NHL is increased by congenital or acquired immunologic defects (Kinlen, 1982; Penn, 1983). Patients with genetically determined immunosuppression, as well as patients iatrogenically immunosuppressed after organ transplantation, have an incidence of NHL and of other lymphoid neoplasias 30 to 50-fold higher than that observed in the general population (Kinlen, 1982; Penn, 1983).

The incidence of NHL in the United States and in other Western countries has been steadily rising since the early 1970s (Pickle *et al.*, 1987), and in more recent years a significant proportion of this increase is attributable to NHL that arise in the setting of human immunodeficiency virus (HIV) infection (Karp & Broder, 1991).

Acquired immunodeficiency syndrome (AIDS)-associated NHL is characteristically a high-grade B-cell malignancy, often multifocal, that frequently originates in extra-nodal sites, in particular in the central nervous system (Ziegler *et al.*, 1984; Kaplan *et al.*, 1989). Interestingly, Epstein Barr virus (EBV) infection is consistently present in primary lymphomas of the central nervous system among AIDS patients (MacMahon *et al.*, 1991), and also many of the AIDS-associated lymphomas classified as immunoblastic or Burkitt's lymphomas appear to be EBV-driven lymphoproliferations (Editorial, 1991).

As of June 1991, 53,042 AIDS cases, from 21 countries belonging to the World Health Organization (WHO) European Region, were reported to the WHO Collaborating Centre on AIDS in Paris, France. These data were used in order to describe the epidemiology of AIDS-associated NHL in the different HIV transmission categories and geographic areas, thus extending and updating a previous study on a more limited number of cases in 15 European countries (Casabona *et al.*, 1991).

Correspondence: D. Serraino, Epidemiology Unit, Aviano Cancer Centre, Via Pedemontana Occ., 33081 Aviano (PN), Italy.
Received 24 March 1992; and in revised form 5 June 1992.

### Material and methods

The 30th June 1991 update of the European Non Aggregate AIDS Data Set was analysed (European Centre for the Epidemiological Monitoring of AIDS, 1991). Such data set contains information for each individual AIDS case recorded by the national surveillance system of 21 countries (Table I). The data are collected by the WHO Collaborating Centre on AIDS in Paris, France, according to a standard core of epidemiologic information, which includes country of report, sex, age, year of AIDS diagnosis, HIV transmission category and reported indicator disease at the time of AIDS diagnosis.

Disease indicators were classified according to the following hierarchy: opportunistic infections; Kaposi's sarcoma (KS); opportunistic infections plus KS; all NHL (i.e., immunoblastic lymphoma, Burkitt's lymphoma and primary lymphoma of the brain); HIV encephalopathy; HIV wasting syndrome and lymphoid interstitial pneumonia. Whereas primary lymphoma of the brain and Burkitt's lymphoma were included in the Center for Disease Control (CDC) AIDS definition criteria since 1981, immunoblastic lymphoma has been a reportable condition only since 1985 (Centers for Disease Control, 1987). Cases showing more than one disease indicator at the time of AIDS diagnosis were assigned to the one which ranked first in the aforementioned classification.

Individuals were assigned to HIV transmission category according to CDC defined criteria. The effects of age, sex, year of AIDS diagnosis and HIV transmission category on the frequency of AIDS-associated NHL were modelled by use of unconditional multiple logistic regression equations, fitted by the method of maximum likelihood (Baker & Nelder, 1978; Breslow & Day, 1980). In the assessment of the influence of age on the proportion of AIDS cases with NHL, an interaction term for age and HIV transmission category (i.e., homosexuals, bisexuals, heterosexuals versus drug users) was included in the model. The significance of linear trends was assessed by computing the difference between the deviances of the model with and without the variable of interest (Baker, 1985).

**Table I** Distribution of AIDS cases by country of residence and non-Hodgkin's lymphoma at presentation. WHO European Region, 1982–1991

| *Country of residence* | *Number of AIDS cases* | *AIDS cases with non-Hodgkin's lymphoma* | | |
|---|---|---|---|---|
| | No. | No. | (%) | MLR-OR[a] (95% CI) |
| Northern Europe: | | | | |
| Germany | 6,604 | 241 | (3.6) | |
| Iceland | 18 | 0 | (0.0) | |
| Netherlands | 1,745 | 65 | (3.7) | |
| Norway | 217 | 5 | (2.3) | |
| Sweden | 587 | 26 | (4.4) | |
| United Kingdom | 4,758 | 140 | (2.9) | |
| Total | 13,929 | 477 | (3.4) | 1[b] |
| Central Europe: | | | | |
| Austria | 594 | 32 | (5.4) | |
| Belgium | 896 | 30 | (3.3) | |
| France | 15,534 | 481 | (3.1) | |
| Monaco | 6 | 0 | (0.0) | |
| Switzerland | 1,891 | 47 | (2.5) | |
| Total | 18,921 | 590 | (3.1) | 1.0 (0.9–1.1) |
| Southern Europe: | | | | |
| Israel | 153 | 7 | (4.6) | |
| Italy | 9,792 | 313 | (3.2) | |
| Portugal | 676 | 17 | (2.5) | |
| Spain | 9,112 | 202 | (2.2) | |
| Turkey | 44 | 0 | (0.0) | |
| Total | 19,777 | 539 | (2.7) | 0.9 (0.8–1.0) |
| Eastern Europe: | | | | |
| Czechoslovakia | 25 | 1 | (4.0) | |
| Hungary | 59 | 3 | (5.1) | |
| Poland | 70 | 0 | (0.0) | |
| USSR | 50 | 0 | (0.0) | |
| Yugoslavia | 211 | 7 | (3.3) | |
| Total | 415 | 11 | (2.7) | 0.8 (0.4–1.4) |
| All countries | 53,042 | 1,617 | (3.0) | |

[a]Multiple logistic regression (MLR) odds ratio (OR) and 95% confidence interval (CI), adjusted for age, sex, HIV transmission category and year of AIDS diagnosis. [b]Reference category.

### Results

NHL as disease indicator at AIDS diagnosis was present in 1,617 out of the 53,042 cases (3.0%) from the WHO European region, reported to the WHO-Collaborating Centre on AIDS in Paris, France, as of the end of June 1991.

As shown in Table I, the percentage of AIDS cases presenting with NHL ranged from 0.0% in Iceland, Monaco, Poland, USSR and Turkey to 5.4% in Austria (Table I). Overall, Eastern and Southern European countries showed a lower percentage of NHL (2.7% in both areas) among their AIDS cases than Central (3.1%) and Northern (3.4%) countries (Table I). In comparison with Northern Europe, and after allowance for the effect of age, sex, HIV transmission category and year of AIDS diagnosis, however, no significant differences emerged (Table I).

Over time, AIDS-associated NHL in the WHO European region increased very substantially in absolute terms in all transmission categories (from 16 in 1984 to 398 in 1990) (Figure 1). As a proportion of AIDS cases, NHL did not show a significant change (2.9% up to 1985 verses 3.1% in 1990–91). An upward trend was, however, detected in homosexual or bisexual men (9.7% per year; 95% confidence interval CI: 4.7%; 14.9%). Conversely, a significant downward trend in the percentage of AIDS cases presenting with NHL emerged among intravenous drug users (IVDUs) (– 17.1% per year; 95% CI: – 22.2%; – 11.6%) (Figure 2).

As concerns HIV transmission category, the frequency of NHL ranged from 1.1% in children infected perinatally to 3.9% among haemophiliacs (Table II). Odds ratios (ORs) of presenting NHL at AIDS diagnosis were computed taking IVDUs as reference category. After allowance for the effect of age, sex and year of AIDS diagnosis, an excess of borderline statistical significance was found among homosexual or bisexual men (OR = 1.2, 95% CI: 1.0–1.3). Conversely, a marked lack of AIDS-associated NHL appeared among children infected perinatally (OR = 0.4, 95% CI: 0.2–0.7) (Table II).

Less than 1% of AIDS cases in the first year of life had NHL. Such percentage peaked up to nearly 4% in the 10–19

**Figure 1** Number of AIDS cases with non-Hodgkin's lymphoma (NHL) by year of AIDS diagnosis and transmission category. Europe, 1982–1991.

**Figure 2** Percent of AIDS cases with non-Hodgkin's lymphoma (NHL) by year of AIDS diagnosis and transmission category. WHO European Region, 1982–1991.

**Table II** Distribution of AIDS cases with non-Hodgkin's lymphoma by transmission category. WHO European Region, 1982–1991[a]

| | *Number of AIDS cases* | *AIDS cases with non-Hodgkin's lymphoma* | | |
|---|---|---|---|---|
| | No. | No. | (%) | MLR-OR[b] (95% CI) |
| *Transmission catetory* | | | | |
| IVDUs[c] | 17,695 | 467 | (2.6) | 1[d] |
| Homosexual men | 23,275 | 791 | (3.4) | 1.2 (1.0–1.3) |
| Homosexual men and IVDUs | 1,012 | 22 | (2.2) | 0.8 (0.5–1.2) |
| Heterosexuals | 4,487 | 116 | (2.6) | 1.0 (0.8–1.2) |
| Haemophiliacs | 1,422 | 55 | (3.9) | 1.3 (0.9–1.7) |
| Blood transfusion recipients | 1,645 | 52 | (3.2) | 1.1 (0.8–1.5) |
| Mother to child | 885 | 10 | (1.1) | 0.4 (0.2–0.7) |

[a]It does not include 2,621 cases with unknown HIV transmission category. [b]Multiple logistic regression (MLR) odds ratio (OR) and 95% confidence interval (CI), adjusted for age, sex and year of AIDS diagnosis. [c]Intravenous drug users. [d]Reference category.

age group, to decrease steadily thereafter until the age group 30–39. A second, more elevated peak (4.3%) was seen at age 50–59 (Figure 3). On account of small figures, the percentages of AIDS cases with NHL in younger age groups had, however, very wide 95% CIs (Figure 3).

The age pattern of NHL showed discrepancies across HIV transmission categories. The percentage of AIDS cases presenting with NHL tended to increase with increasing age among homosexual or bisexual men and heterosexuals, whereas a significant inverse trend was seen among IVDUs (Table III). The heterogeneity of these two trends was significant ($\chi^2_1 = 31.11$; $P < 0.001$).

NHL was diagnosed slightly more frequently among IVDU men (2.8%) than in IVDU women (2.1%) (OR for women *vs* men = 0.7, 95% CI: 0.6–0.9). No difference, on the other hand, emerged between the frequency of NHL in heterosexual men (2.5%) and in heterosexual women (2.7%) (Table III).

### Discussion

The present paper is the largest report on a series of AIDS-associated NHL outside the United States (Beral *et al.*, 1991) published so far. In agreement with data from the United

**Figure 3** Percent of AIDS cases with non-Hodgkin's lymphoma (NHL) by age and 95% confidence intervals. WHO European Region, 1982–1991. (In parenthesis, number of AIDS cases with NHL).

**Table III** Distribution of AIDS cases with non-Hodgkin's lymphoma (NHL) by age, transmission category and sex. Europe, 1982–1991.

| | *Transmission category* | | | | | | | | |
|---|---|---|---|---|---|---|---|---|---|
| | *Homosexual men* | | | *Intravenous drug users* | | | *Heterosexuals* | | |
| | AIDS cases:NHL | (%) | MLR-OR[a] (95% CI) | AIDS cases:NHL | (%) | MLR-OR[a] (95% CI) | AIDS cases:NHL | (%) | MLR-OR[a] (95% CI) |
| Age (years):[b] | | | | | | | | | |
| <20 | 31:1 | (3.2) | 1[c] | 113:3 | (2.7) | 1[c] | 33:0 | (0.0) | 1[c] |
| 20–24 | 799:24 | (3.0) | | 2,755:106 | (3.9) | | 382:10 | (2.6) | |
| 25–29 | 3,176:108 | (3.4) | 1.1 (0.7–1.7) | 7,777:203 | (2.6) | 0.7 (0.6–0.9) | 952:20 | (2.1) | 0.9 (0.4–1.9) |
| 30–34 | 4,706:130 | (2.8) | 0.9 (0.6–1.4) | 4,910:104 | (2.1) | 0.6 (0.5–0.8) | 971:14 | (1.4) | 0.6 (0.3–1.4) |
| 35–39 | 4,825:134 | (2.8) | 0.9 (0.6–1.4) | 1,599:39 | (2.4) | 0.7 (0.5–1.2) | 650:20 | (3.1) | 1.4 (0.6–3.1) |
| 40–49 | 6,758:259 | (3.8) | 1.3 (0.8–1.9) | 416:11 | (2.6) | 0.7 (0.4–1.2) | 826:25 | (3.0) | 1.4 (0.7–3.0) |
| 50–59 | 2,381:107 | (4.5) | 1.5 (0.9–2.3) | 44:0 | (0.0) | | 450:18 | (4.0) | 1.9 (0.8–4.2) |
| > = 60 | 543:23 | (4.2) | 1.4 (0.8–2.4) | 5:0 | (0.0) | | 213:7 | (3.3) | 1.3 (0.5–3.3) |
| $\chi^2_1$ for trend | | 13.11;p<0.001 | | | 8.00;p = 0.005 | | | 9.70;p = 0.002 | |
| Sex: | | | | | | | | | |
| Men | 23,275:791 | (3.4) | | 13,768:383 | (2.8) | 1[c] | 2,548:64 | (2.5) | 1[c] |
| Women | | | | 3,927:84 | (2.1) | 0.7 (0.6–0.9) | 1,939:52 | (2.7) | 1.2 (0.8–1.8) |

[a]Multiple logistic regression (MLR) odds ratio (OR) and 95% confidence interval (CI), allowing for age and sex (when appropriate), and year of AIDS diagnosis. [b]The sum does not add up to the total because of missing values. [c]Reference category.

States, where 2,824 out of 97,258 AIDS cases (2.9%) had NHL (Beral *et al.*, 1991), and with a similar study on AIDS-associated NHL in 15 European countries, where 3.3% of cases had NHL (Casabona *et al.*, 1991), the results of the present study indicate that NHL is associated with 3.0% of AIDS cases in the WHO European Region. This figure is also similar to that found in other immunosuppressed groups not infected with HIV (Fraumeni & Hoover, 1977; Penn, 1986).

Studies based on AIDS surveillance data tend, however, to substantially underestimate the real proportion of AIDS patients who develop NHL. The occurrence of NHL is rarely the presenting disease in a patient with AIDS; most often NHL is diagnosed after the development of KS or opportunistic infections or it may even be silent during life (Myskowski *et al.*, 1990). In a series of 43 patients with AIDS-associated NHL from Memorial Hospital (Lowenthal *et al.*, 1988), one third presented initially with KS and one third had an antecedent opportunistic infection. Furthermore, in seven out of nine patients with primary lymphoma of the brain the diagnosis was made at autopsy only (Lowenthal *et al.*, 1988). Clinical evidence suggests that approximately 10% of patients with AIDS develop NHL during the course of their disease (Kaplan *et al.*, 1989; Cremer *et al.*, 1990; Monfardini *et al.*, 1990), but for HIV infected patients treated with long-term antiretroviral therapy such percentage seems to increase even more, being nearly 45% after 3 years of treatment (Pluda *et al.*, 1990).

As already reported by Casabona *et al.* (1991), also in the present study the proportions of AIDS cases with NHL were quite comparable in the different areas of Europe. The discrepant figures derived, however, only from countries (i.e., Eastern and Southern Europe) where the AIDS epidemic is still in a very early phase, and/or the accuracy in NHL diagnoses may be low (Jensen *et al.*, 1990).

In the United States, IVDUs with AIDS had approximately half the probability of presenting with NHL as compared to homosexual or bisexual men (Beral *et al.*, 1991). Accordingly, in the present study the frequency of NHL was higher in homosexual or bisexual men and haemophiliacs than in IVDUs, but such differences were smaller than in the United States.

It is possible to hypothesise that the different organisation of the health care system in most European countries, as compared to the United States, may allow a more homogenous access to the diagnostic procedures across the various strata of the population, including IVDUs, thus reducing artifactual differences. Indeed, the percentages of AIDS cases with NHL among homosexual or bisexual men in the United States and Europe were identical (3.4%), whereas a remarkable excess of NHL among European IVDUs was seen (2.6% *vs* 1.6%). An alternative explanation of this discrepancy between data from Europe and from the United States may thus be a lower predisposition toward the development of NHL (or a stronger tendency to develop opportunistic infections or KS) in North American IVDUs, who are in a substantial proportion black.

As concerns the proportion of AIDS-associated NHL in different age groups, the present data from the WHO European Region and the data from the United States are consistent in showing a bimodal distribution, with a first peak in adolescence (i.e. age 10–19) and the second in middle age (50–59 years). Unfortunately, the information on type of NHL (i.e., immunoblastic lymphoma, Burkitt's lymphoma and brain lymphoma) was not available in the present data set, thus hampering the interpretation of this age-related behaviour, which is partly different from that recorded in the age curve of non AIDS-associated NHL (Pickle *et al.*, 1987; World Health Organization, 1990). According to Beral *et al.* (1991), however, the early peak should be attributed to the early occurrence of Burkitt's lymphoma, while the subsequent increase at older age should chiefly reflect the steady rise of immunoblastic lymphoma with age.

Two potential differences in AIDS-associated NHL according to HIV transmission categories (i.e., sexual versus parenteral transmission) emerged for the first time from the present study. The frequency of AIDS cases presenting with NHL seemed to decline over the examined calendar period and across subsequent age groups in IVDUs, but not in homosexual or bisexual men and in heterosexuals. The study of such discrepant patterns is made difficult by the substantial differences in both the geographic and age distribution between the largest HIV transmission categories. At this regard, a certain excess of IVDUs in Southern European countries, more lately involved by the AIDS epidemic than Northern countries (European Centre for the Epidemiological Monitoring of AIDS, 1991) and the lack of middle age individuals among IVDUs is worth noting.

The relative increase of NHL as AIDS-indicator disease among homosexual or bisexual men over the calendar period and age may mirror the parallel decrease in KS, but other possible explanations (e.g., differential changes in the frequency of competing indicator diseases other than KS or in diagnostic accuracy over time and/or age) are also worth considering.

These differences in the distribution of AIDS-associated NHL between IVDUs and homosexual or bisexual men must be interpreted with utmost caution, on account of the aforementioned limitations of the reporting system. We think, however, that they deserve further attention and investigation, possibly by means of datasets which include information on the follow up of AIDS patients and the histologic types of NHL.

We wish to thank the national correspondents of the 21 countries who provided data for the European Non Aggregate AIDS Data Set: Austria (Federal Ministry of Health and Environmental Protection, Vienna); Belgium (Conseil Supérieur pour la Coordination de la Lutte contre le SIDA, Institut d'Hygiène et d'Epidémiologie, Brussels); Czechoslovakia (Institute of Hygiene and Epidemiology, Prague); France (Direction Général de la Santé, Paris); Germany, F.R. (AIDS Zentrum im Bundesgesunheitsamt, Berlin); Hungary (National Institute of Hygiene, Budapest); Iceland (General Direction of Public Health, Reykjavik); Israel (Ministry of Health, Jerusalem); Italy (Ministry of Health, Rome); Monaco (Direction de l'Action Sanitaire et Sociale, Monaco); Netherlands (Staatstoezicht op de Volksgezondheid, Rijswijk); Norway (National Institute of Public Health, Oslo); Poland (National Institute of Hygiene, Warsaw); Portugal (Instituto Nacional de Saude, Lisbon); Spain (Instituto de Salud 'Carlos III', Madrid); Sweden (National Bacteriological Laboratory, Stockholm); Switzerland (Officer Fédéral de la Santé Publique, Liebefeld); Turkey (Ministry of Health and Social Assistance, Ankara); United Kingdom (Communicable Disease Surveillance Centre, London); USSR (Ministry of Health of the USSR, Moscow); Yugoslavia (Federal Institute of Public Health, Belgrade).

This work was supported by a grant from the Ministero della Sanità - Istituto Superiore di Sanità, IV Progetto AIDS, contract 6202-027 and contract 6205-006 - Rome, Italy. It was undertaken during the tenure of a Research Fellowship, awarded by the Ministero della Sanità - Istituto Superiore di Sanità, Rome, Italy - to Dr Giuseppe Salamina.

### References

BAKER, R.J. & NELDER, J.A. (1978). *The GLIM system, Release 3.* Numerical Algorithms Group, Oxford.

BERAL, V., PETERMAN, T., BERKELMAN, R. & JAFFE, H. (1991). AIDS-associated non-Hodgkin lymphoma. *Lancet*, **i**, 805–809.

BRESLOW, N.E. & DAY, N.E. (1980). Statistical methods in cancer research. *IARC Sci. Publ.* No. 32 pp. 192–246: Lyon.

CASABONA, J., MELBYE, M., BIGGAR, R. & THE AIDS REGISTRY CONTRIBUTORS (1991). Kaposi's sarcoma and non-Hodgkin's lymphoma in European AIDS cases. No excess risk of Kaposi's sarcoma in mediterranean countries. *Int. J. Cancer*, **47**, 49–53.

CENTERS FOR DISEASE CONTROL (1987). Revision of the CDC surveillance case definition for acquired immune deficiency syndrome. *MMWR*, **36** (Suppl. 1S), 1S–15S.
CREMER, K.J., SPRING, S.B. & GRUBER, J. (1990). Role of human immunodeficiency virus type-1 and other viruses in malignancies associated with acquired immunodeficiency syndrome. *J. Natl Cancer Inst.*, **82**, 1016–1024.
EDITORIAL (1991). Epstein-Barr virus and AIDS-associated lymphomas. *Lancet*, **ii**, 979–981.
EUROPEAN CENTRE FOR THE EPIDEMIOLOGICAL MONITORING OF AIDS (1991). AIDS Surveillance in Europe - *Quarterly Report* No. 30, 30th June 1991. WHO-EC Collaborating Centre on AIDS: Paris.
FRAUMENI, J.F. & HOOVER, R. (1977). Immunosurveillance and cancer: epidemiologic evidence. *NCI Monogr.*, **47**, 121–126.
JENSEN, O.M., ESTEVE, J., MOLLER, H. & RENARD, H. (1990). Cancer in the European Community and its member states. *Eur. J. Cancer*, **26**, 1167–1256.
KAPLAN, L.D., ABRAMS, D.I., FEIGAL, E., MCGRATH, M., KAHN, J., NEVILLE, P., ZIEGLER, J. & VOLBERDING, P.A. (1989). AIDS-associated non-Hodgkin's lymphoma in San Francisco. *J. Amer. Med. Ass.*, **261**, 719–724.
KARP, J.E. & BRODER, S. (1991). Acquired immunodeficiency syndrome and non-Hodgkin's lymphomas. *Cancer Res.*, **51**, 4743–4756.
KINLEN, L.J. (1982). Immunosuppressive therapy and cancer. *Cancer Surv.*, **1**, 567–583.
LEVI, F., MAISONNEUVE, P., FILIBERTI, R., LA VECCHIA, C. & BOYLE, P. (1989). Cancer incidence and mortality in Europe. *Soz. Praventivmed.*, **34** (Suppl. 2), S54–S55.
LOWENTHAL, D.A., STRAUS, D.J., CAMPBELL, W.S., GOLD, J.W.M., CLARKSON, B.D. & KOZINER, B. (1988). AIDS-related lymphoid neoplasia. The Memorial Hospital experience. *Cancer*, **61**, 2325–2337.
MACMAHON, E.M.E., GLASS, J.D., HAYWARD, S.D., MANN, R.B., BECKER, P.S., CHARACHE, P., MCARTHUR, J.C. & AMBINDER, R.F. (1991). Epstein-Barr virus in AIDS-related primary central nervous system lymphoma. *Lancet*, **ii**, 969–973.
MONFARDINI, S., VACCHER, E., FOA', R., TIRELLI, U., AND OTHERS FOR THE ITALIAN COOPERATIVE GROUP ON AIDS-RELATED TUMORS (1990). AIDS-associated non-Hodgkin's lymphoma in Italy: intravenous drug users versus homosexual men. *Ann. Oncol.*, **1**, 203–211.
MYSKOWSKI, P.L., STRAUS, D.J. & SAFAI, B. (1990). Lymphoma and other HIV-associated malignancies. *J. Am. Acad. Dermatol.*, **22**, 1253–1260.
PENN, I. (1983). Lymphomas complicating organ transplantation. *Transplant Proc.*, **15**, 2790–2797.
PENN, I. (1986). The occurrence of malignant tumours in immunosuppressed states. *Progr. Allergy*, **37**, 259–300.
PICKLE, L.W., MASON, T.J., HOWARD, N., HOOVER, R. & FRAUMENI, J.F. (1987). *Atlas of United States cancer mortality among whites: 1950–1980*. US Department of Health and Human Services, Public Health Service, National Institute of Health, DHHS Publication No. (NIH) 87-2900 p 182: Washington, DC.
PLUDA, J.M., YARCHOAN, R., JAFFE, E.S., FEUERSTEIN, I.M., SOLOMON, D., STEINBERG, S.M., WYVILL, K.M., RAUBITSCHEK, A., KATZ, D. & BRODER, S. (1990). Development of non-Hodgkin's lymphoma in a cohort of patients with severe human immunodeficiency virus (HIV) infection on long term antiretroviral therapy. *Ann. Intern. Med.*, **113**, 276–282.
WORLD HEALTH ORGANIZATION (1990). *Patterns of cancer in five continents*. Whelan, S.L., Parkin, D.M. & Masuyer, E. (eds), IARC Sci. Publ. No. 102 pp 154–155: Lyon.
ZIEGLER, J.L., BECKSTEAD, A.J., VOLBERDING, P.A., ABRAMS, D.I., LEVINE, A.M., LUKES, R.J., GILL, P.S., BURKES, R.L., MEYER, P.R., METROKA, G.E., MOURADIAN, J., MOORE, A., RIGGS, S.A., BUTLER, J.J., CABALLINAS, F.C., HERSH, E., NEWELL, G.R., LAUBENSTEIN, L.J., KNOWLES, D., ODAJNYK, C., RAPHAEL, B., KOZINER, B., URMACHER, C. & CLARKSON, B.D. (1984). Non-Hodgkin's lymphoma in 90 homosexual men. Relation to generalized lymphadenopathy and the acquired immunodeficiency syndrome. *N. Engl. J. Med.*, **311**, 565–570.


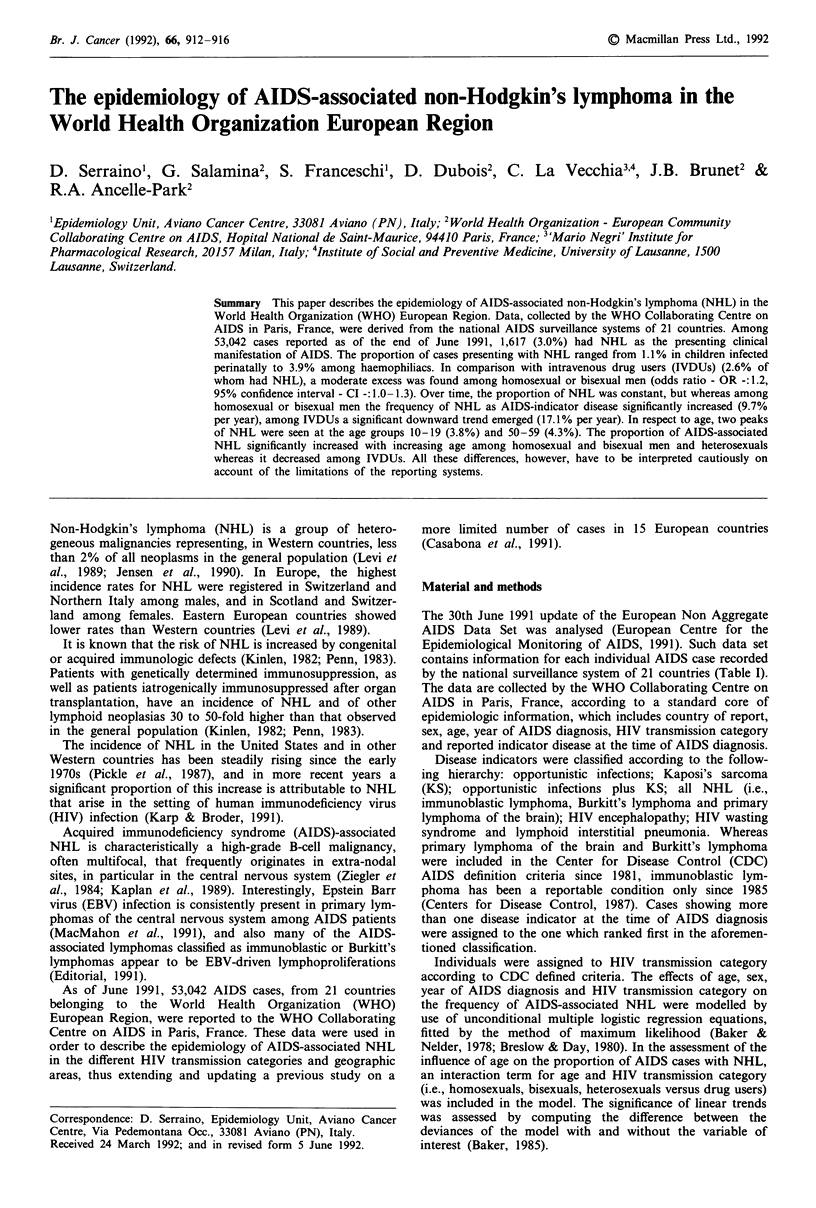

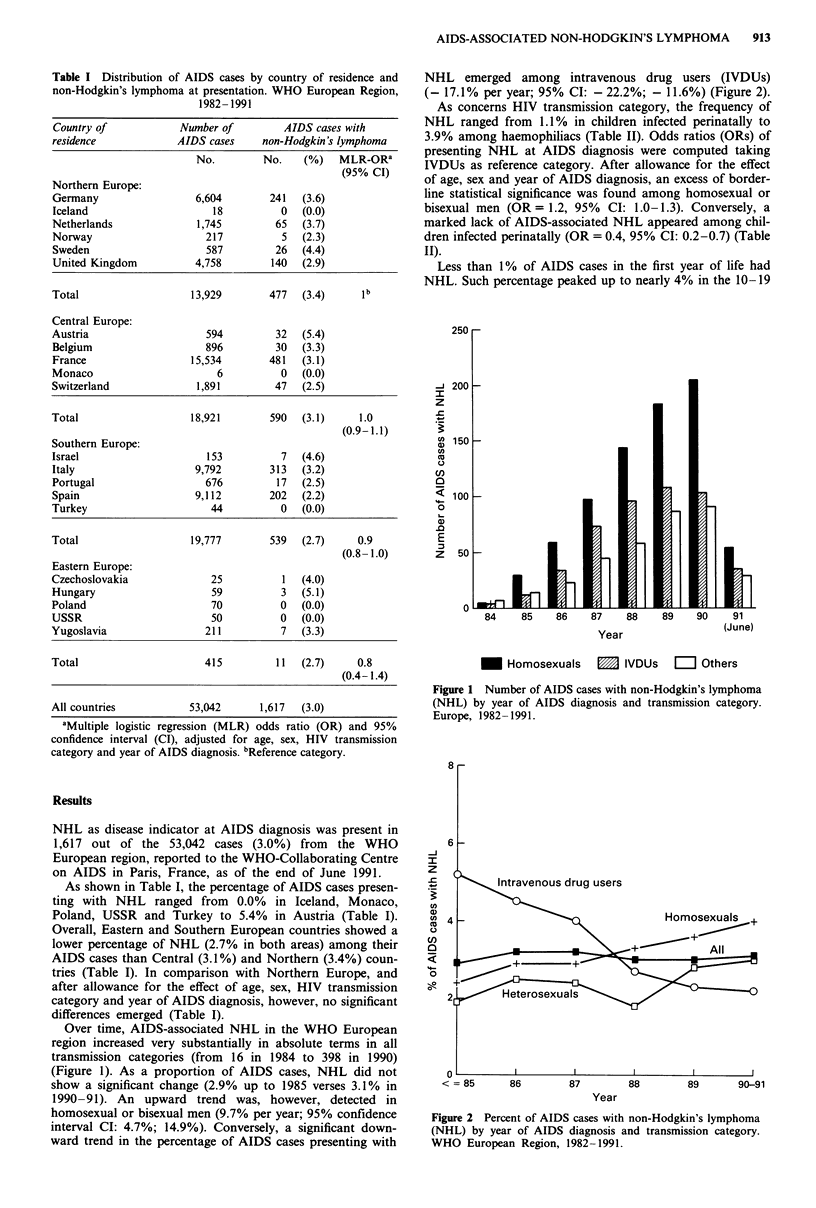

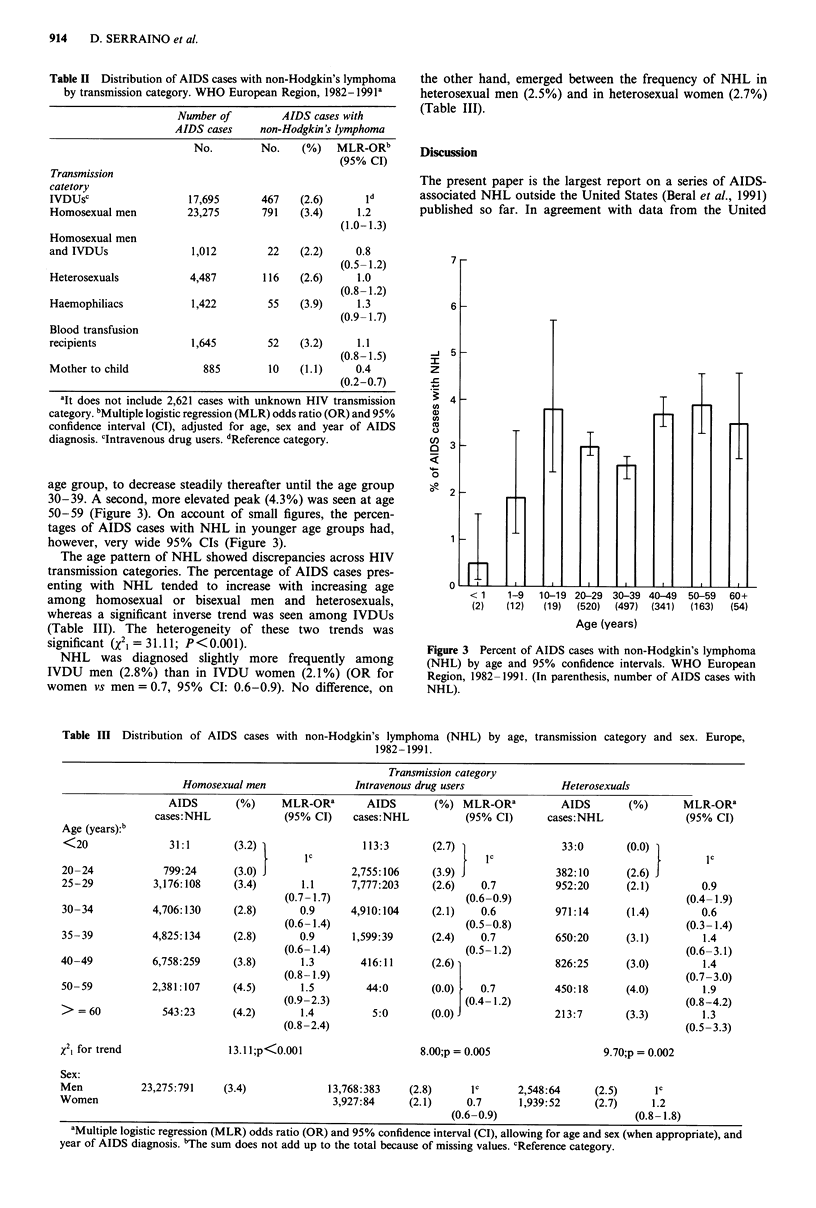

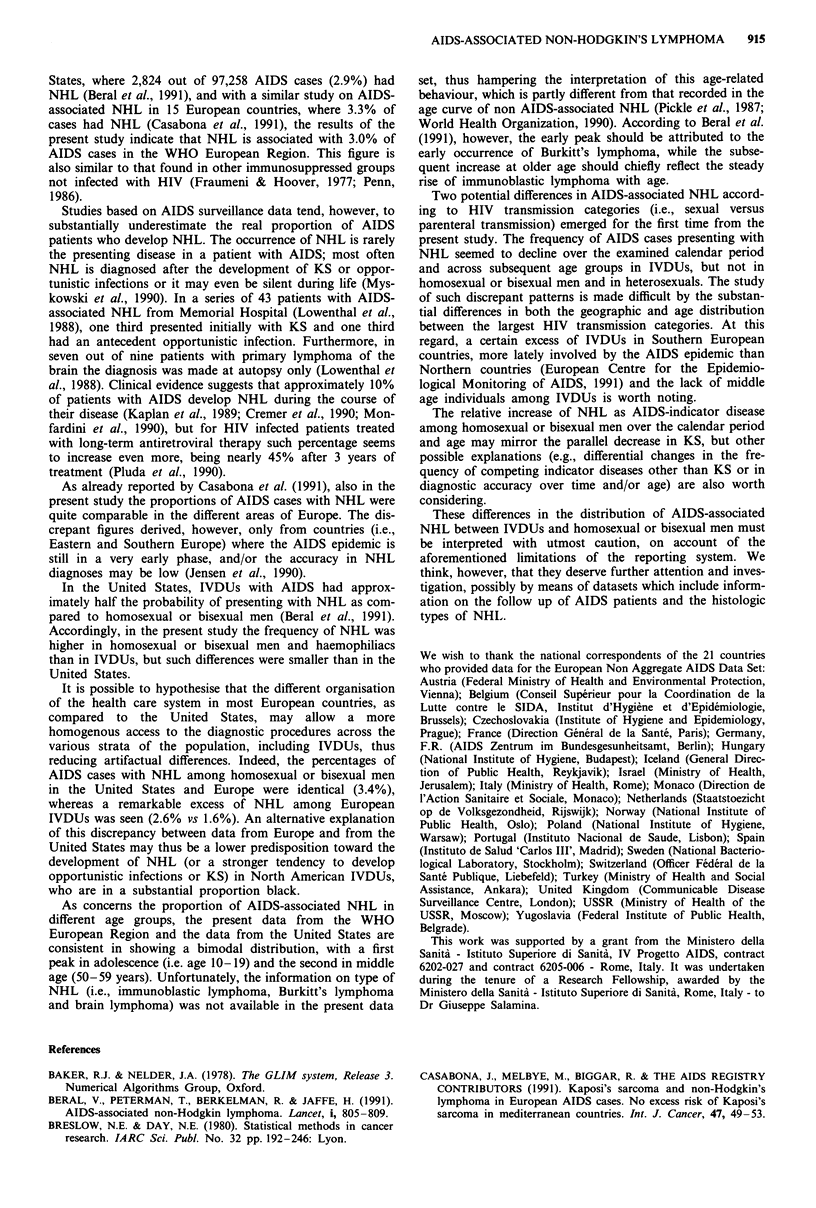

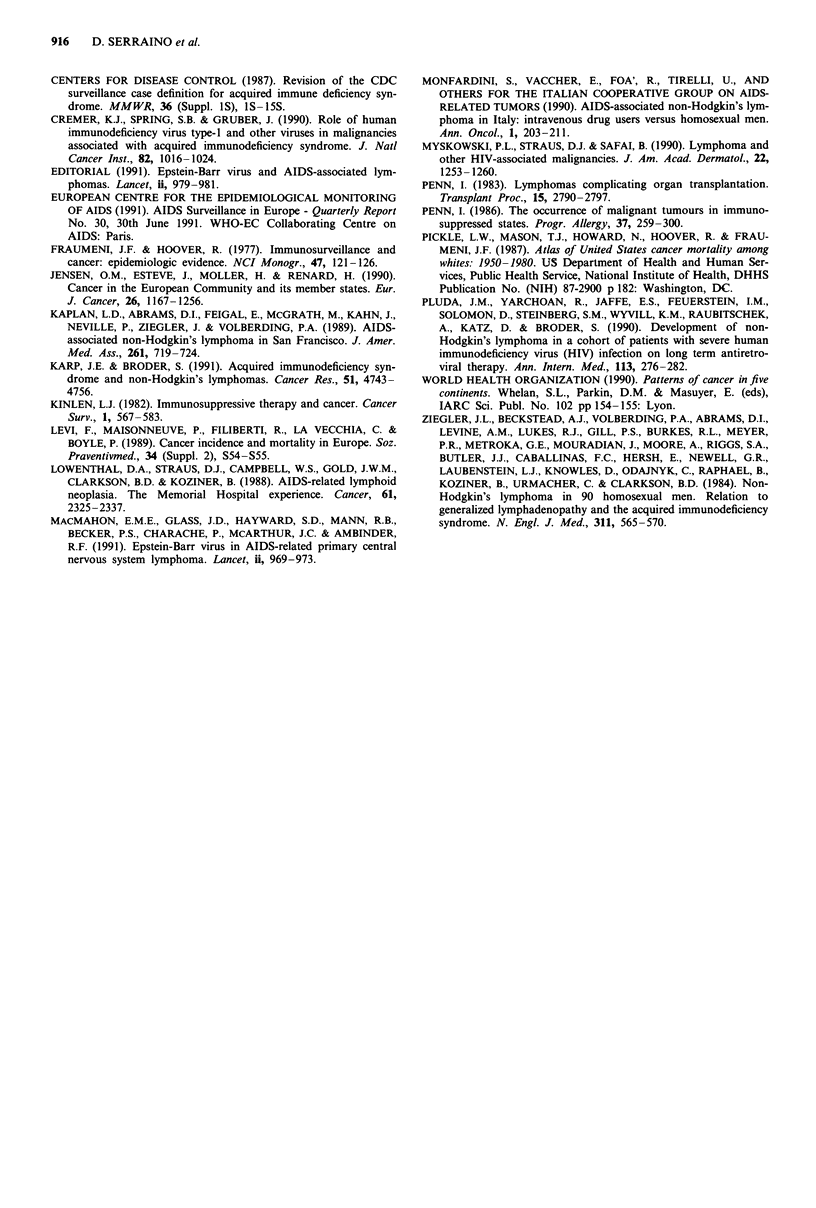


## References

[OCR_00731] Beral V., Peterman T., Berkelman R., Jaffe H. (1991). AIDS-associated non-Hodgkin lymphoma.. Lancet.

[OCR_00739] Casabona J., Melbye M., Biggar R. J. (1991). Kaposi's sarcoma and non-Hodgkin's lymphoma in European AIDS cases. No excess risk of Kaposi's sarcoma in Mediterranean countries.. Int J Cancer.

[OCR_00752] Cremer K. J., Spring S. B., Gruber J. (1990). Role of human immunodeficiency virus type 1 and other viruses in malignancies associated with acquired immunodeficiency disease syndrome.. J Natl Cancer Inst.

[OCR_00768] Fraumeni J. F., Hoover R. (1977). Immunosurveillance and cancer: epidemiologic observations.. Natl Cancer Inst Monogr.

[OCR_00772] Jensen O. M., Estève J., Møller H., Renard H. (1990). Cancer in the European Community and its member states.. Eur J Cancer.

[OCR_00777] Kaplan L. D., Abrams D. I., Feigal E., McGrath M., Kahn J., Neville P., Ziegler J., Volberding P. A. (1989). AIDS-associated non-Hodgkin's lymphoma in San Francisco.. JAMA.

[OCR_00783] Karp J. E., Broder S. (1991). Acquired immunodeficiency syndrome and non-Hodgkin's lymphomas.. Cancer Res.

[OCR_00797] Lowenthal D. A., Straus D. J., Campbell S. W., Gold J. W., Clarkson B. D., Koziner B. (1988). AIDS-related lymphoid neoplasia. The Memorial Hospital experience.. Cancer.

[OCR_00803] MacMahon E. M., Glass J. D., Hayward S. D., Mann R. B., Becker P. S., Charache P., McArthur J. C., Ambinder R. F. (1991). Epstein-Barr virus in AIDS-related primary central nervous system lymphoma.. Lancet.

[OCR_00809] Monfardini S., Vaccher E., Foà R., Tirelli U. (1990). AIDS-associated non-Hodgkin's lymphoma in Italy: intravenous drug users versus homosexual men. The Italian Cooperative Group on AIDS-Related Tumors (GICAT).. Ann Oncol.

[OCR_00816] Myskowski P. L., Straus D. J., Safai B. (1990). Lymphoma and other HIV-associated malignancies.. J Am Acad Dermatol.

[OCR_00825] Penn I. (1986). The occurrence of malignant tumors in immunosuppressed states.. Prog Allergy.

[OCR_00836] Pluda J. M., Yarchoan R., Jaffe E. S., Feuerstein I. M., Solomon D., Steinberg S. M., Wyvill K. M., Raubitschek A., Katz D., Broder S. (1990). Development of non-Hodgkin lymphoma in a cohort of patients with severe human immunodeficiency virus (HIV) infection on long-term antiretroviral therapy.. Ann Intern Med.

[OCR_00849] Ziegler J. L., Beckstead J. A., Volberding P. A., Abrams D. I., Levine A. M., Lukes R. J., Gill P. S., Burkes R. L., Meyer P. R., Metroka C. E. (1984). Non-Hodgkin's lymphoma in 90 homosexual men. Relation to generalized lymphadenopathy and the acquired immunodeficiency syndrome.. N Engl J Med.

